# Advancing endovascular neurosurgery training with extended reality: opportunities and obstacles for the next decade

**DOI:** 10.3389/fsurg.2024.1440228

**Published:** 2024-08-27

**Authors:** Shray A. Patel, Michael M. Covell, Saarang Patel, Sandeep Kandregula, Sai Krishna Palepu, Avi A. Gajjar, Oleg Shekhtman, Georgios S. Sioutas, Ali Dhanaliwala, Terence Gade, Jan-Karl Burkhardt, Visish M. Srinivasan

**Affiliations:** ^1^ Sidney Kimmel Medical College, Thomas Jefferson University, Philadelphia, PA, United States; ^2^ School of Medicine, Georgetown University, Washington, DC, United States; ^3^ Department of Neurosurgery, Perelman School of Medicine, University of Pennsylvania, Philadelphia, PA, United States; ^4^ Department of Radiology, Perelman School of Medicine, University of Pennsylvania, Philadelphia, PA, United States

**Keywords:** virtual reality, augmented reality, mixed reality, endovascular, neurosurgery

## Abstract

**Background:**

Extended reality (XR) includes augmented reality (AR), virtual reality (VR), and mixed reality (MR). Endovascular neurosurgery is uniquely positioned to benefit from XR due to the complexity of cerebrovascular imaging. Given the different XR modalities available, as well as unclear clinical utility and technical capabilities, we clarify opportunities and obstacles for XR in training vascular neurosurgeons.

**Methods:**

A systematic review following the Preferred Reporting Items for Systematic Reviews and Meta-Analyses (PRISMA) guidelines was conducted. Studies were critically appraised using ROBINS-I.

**Results:**

19 studies were identified. 13 studies used VR, while 3 studies used MR, and 3 studies used AR. Regarding specific educational applications, VR was used for simulation in 10 studies and anatomical modeling in 3 studies. AR was only used for live intra-operative guidance (*n* = 3 studies). MR was only used for modeling and intra-operative teaching. Considering disease-specific uses, XR enhanced trainee understanding of intracranial aneurysms (*n* = 12 studies) and stroke (*n* = 7). XR trained surgeons in diverse neurosurgical procedures, including aneurysm coiling (*n* = 5 studies), diagnostic angiography (*n* = 5), and thrombectomy (*n* = 5).

**Conclusions:**

Anatomical modeling with VR and MR enhances neurovascular anatomy education with patient-specific, 3-D models from imaging data. AR and MR enable live intra-operative guidance, allowing experienced surgeons to remotely instruct novices, potentially improving patient care and reducing geographic disparities. AR overlays enhance instruction by allowing the surgeon to highlight key procedural aspects during training. Inaccurate tracking of surgical tools is an XR technological barrier for modeling and intra-operative training. Importantly, the most reported application of XR is VR for simulation–using platforms like the Mentice VIST and Angio Mentor. 10 studies examine VR for simulation, showing enhanced procedural performance and reduced fluoroscopy use after short training, although long-term outcomes have not been reported. Early-stage trainees benefited the most. Simulation improved collaboration between neurosurgeons and the rest of the surgical team, a promising role in interprofessional teamwork. Given the strength of VR for simulation, MR for simulation is an important gap in the literature for future studies. In conclusion, XR holds promise for transforming neurosurgical education and practice for simulation, but technological research is needed in modeling and intra-procedural training.

## 1 Background

Over the past century, the expansion of neurosurgical approaches has been driven by advancements in imaging technology. Because fluoroscopic imaging in endovascular procedures offers only biplane views, vascular neurosurgery is uniquely positioned to benefit greatly from advanced imaging projections. For example, augmented fluoroscopy—which combines 3-D rotational angiography with 2-D fluoroscopy—has greatly improved endovascular neurosurgery. This progress highlights the potential advantages of further advancements in imaging technology, such as extended reality. Extended reality (XR) is an umbrella term for mixed reality (MR), virtual reality (VR), and augmented reality (AR); it has already found applications across an array of fields, including education, military training, and surgery (1–4).

XR technologies have distinct mechanisms: VR constructs entirely computer-generated environments that change in real-time with input from the surgeon and the physical environment. Instead of projections in a computer-generated environment, AR overlays virtual objects (like 3-D representations of neurovasculature) onto the surgeon's real-world environment. In contrast, MR builds upon AR by allowing physical and virtual elements to interact for example, a surgeon manipulating a 3-D brain model on a table.

What utility might XR provide for neurosurgical training? In a survey of 233 neurosurgeons from 38 countries, 67.4% had simulator access as a trainee, but only 15.3% used it frequently. Notably, the survey responses indicate that the realism and convenience of an improved virtual simulator would increase usage–something newer XR technology makes possible (5, 6). The proposed utility of XR for neurosurgery is broad: one review coins the term “future surgery” to describe the magnitude of XR's impact on a new era of neurosurgery (7, 8). First, XR can create immersive, risk-free environments where trainees can practice complex procedures, improving their skills and confidence before performing on patients. Similarly, XR can also potentially curb expenses and waste in surgical training by offering an alternative to costly simulation environments–reducing the use of cadavers or animal models. Moreover, AR, VR, and MR can provide an enhanced educational experience by improved anatomy visualizations (9, 10). It has been suggested that XR could be useful for tele-proctoring for attendings in remote areas that are not comfortable with advanced vascular procedures, but are the only surgeons available (11, 12). However, while these applications have been proposed, these commentaries lack data-driven evidence that accurately assesses the current state of XR. In a strong state-of-the-art published in *Frontiers in Surgery*, Cannizzaro et al. discuss the great promise that AR holds for neurosurgery. While an extremely robust review, several questions remain unanswered after their work 3 years ago. First, their review only assesses AR, not VR or MR. In addition, their broad approach to general neurosurgery is limited in its ability to provide a detailed analysis of the implications of specific technological advances; this lack of detail is partially because they examine all of neurosurgery, while we will focus on endovascular neurosurgery (13). Given the diversity of neurosurgical procedures, focusing on a subspecialty allows for a standardized collection of outcomes. This focused methodology has been applied to the subspecialty of spinal neurosurgery, but the devices examined in the literature are used for screw placement and discectomy and have little overlap with the devices used in vascular neurosurgery, such as catheters and guidewires (14). Most importantly, among all neurosurgical subspecialties, vascular neurosurgery stands out with the most comprehensive reports in XR, showing significant potential for a systematic review (15).

Here, we aim to provide an unprecedented, detailed, analysis with specific attention to key technical advancements and novel clinical applications of XR for endovascular neurosurgery training. By doing so, we will highlight areas that warrant further study and key strengths to capitalize on, to ultimately facilitate the future integration of AR, VR, and MR into the future of neurosurgery education.

## 2 Materials and methods

This systematic review complied with the Preferred Reporting Items for Systematic Reviews and Meta-Analyses (PRISMA) guidelines (16). The five research databases for this review were PubMed, Scopus, Ovid, EMBASE, and Clarivate Web of Science. The search queries were crafted to cover a comprehensive range of studies related to the use of AR, MR and VR in endovascular neurosurgery. As an example query, we searched PubMed with the following terms: ((vascular neurosurgery[Title/Abstract]) OR (neurovascular[Title/Abstract])) OR (neuro-vascular[Title/Abstract]) OR (neuro-endovascular[Title/Abstract]) OR (interventional neurorad*[Title/Abstract]) OR (neurointervention*[Title/Abstract]) OR (neuro-intervention*[Title/Abstract]) OR (neuro intervention*[Title/Abstract])) AND ((augmented reality[Title/Abstract]) OR (AR[Title/Abstract]) OR (virtual reality[Title/Abstract]) OR (VR[Title/Abstract])). The queries were syntactically modified for the other four databases (Supplementary Data S1). To make sure we did not miss any cutting-edge, recently published studies, our search was last repeated in April 2024.

Next, the titles and abstracts retrieved from these databases were screened for relevance to the research question. Then, articles with ambiguous relevance based on their titles and abstracts were scrutinized further by full text examination. Studies were excluded if they did not mention endovascular neurosurgery, were unrelated to the research question, or were not written in English. Two independent reviewers (S.A.P., M.C.), did all the screening with a third reviewer (S.P.) to resolve conflicts. Reviews and commentaries were excluded so that only papers reporting defined outcomes would be included.

Data was systematically extracted from each selected study, capturing key information such as the type of technology (AR, MR, or VR), its specific application in endovascular neurosurgery, pathologies examined, procedures trained, the experience and education of trainees taught in each study, and the main outcomes and limitations reported. This structured data extraction process facilitated the synthesis of summary figures and enabled a comprehensive comparison of the studies included in the review.

ROBINS-I was used to critically appraise the studies for bias across 7 categories: confounding, selection bias, bias in the classification of interventions, bias due to deviations from the intended interventions, bias due to missing data, bias in the measurement of outcomes, and bias in the selection of reported results (17).

## 3 Results

**Figure 1:**
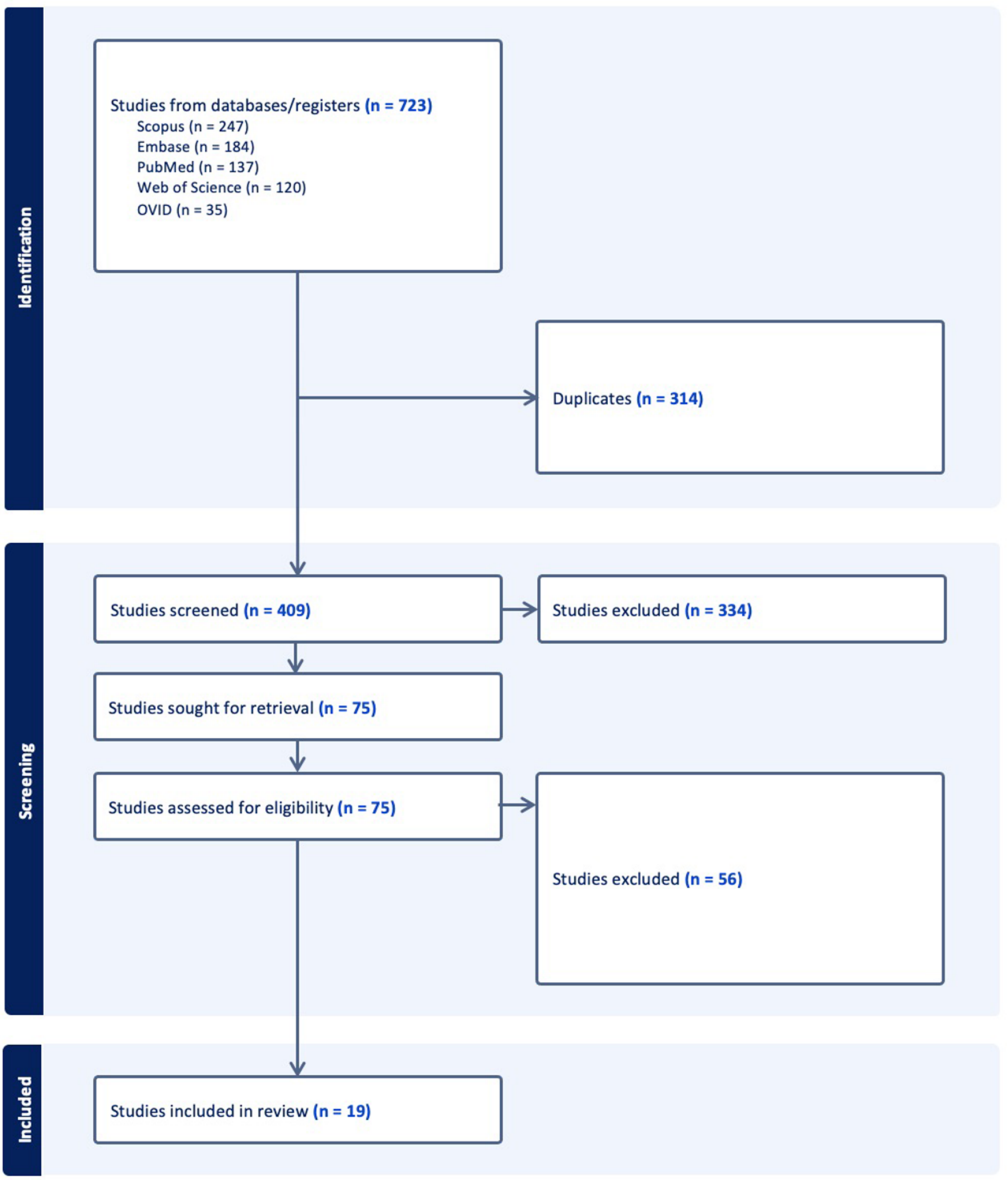
PRISMA flow chart for the literature search.

**Figure 2:**
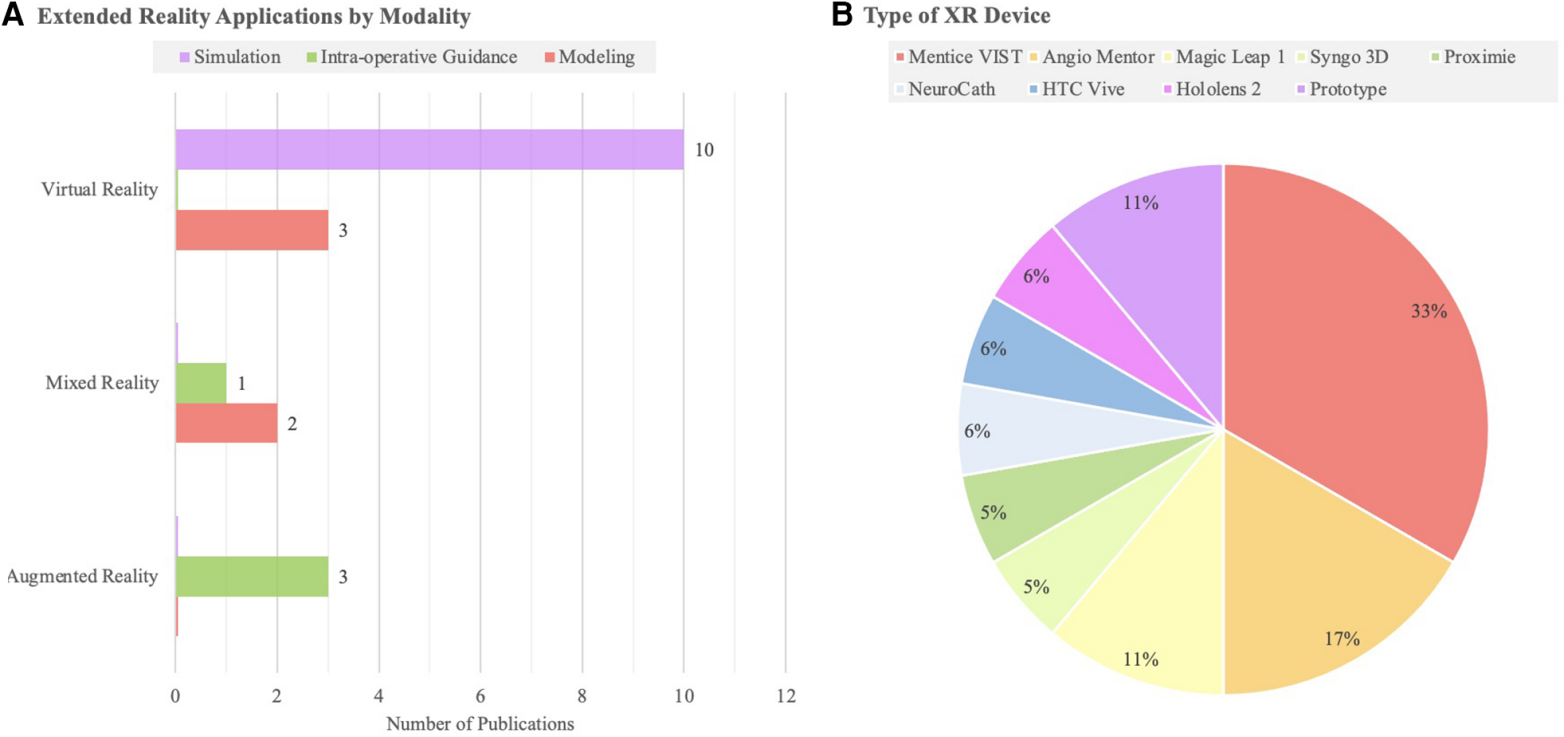
**(A)** A bar chart highlighting the number of studies of each extended reality modality (AR, MR, VR) by educational domain. **(B)** Types of XR devices reported in each study identified.

**Figure 3:**
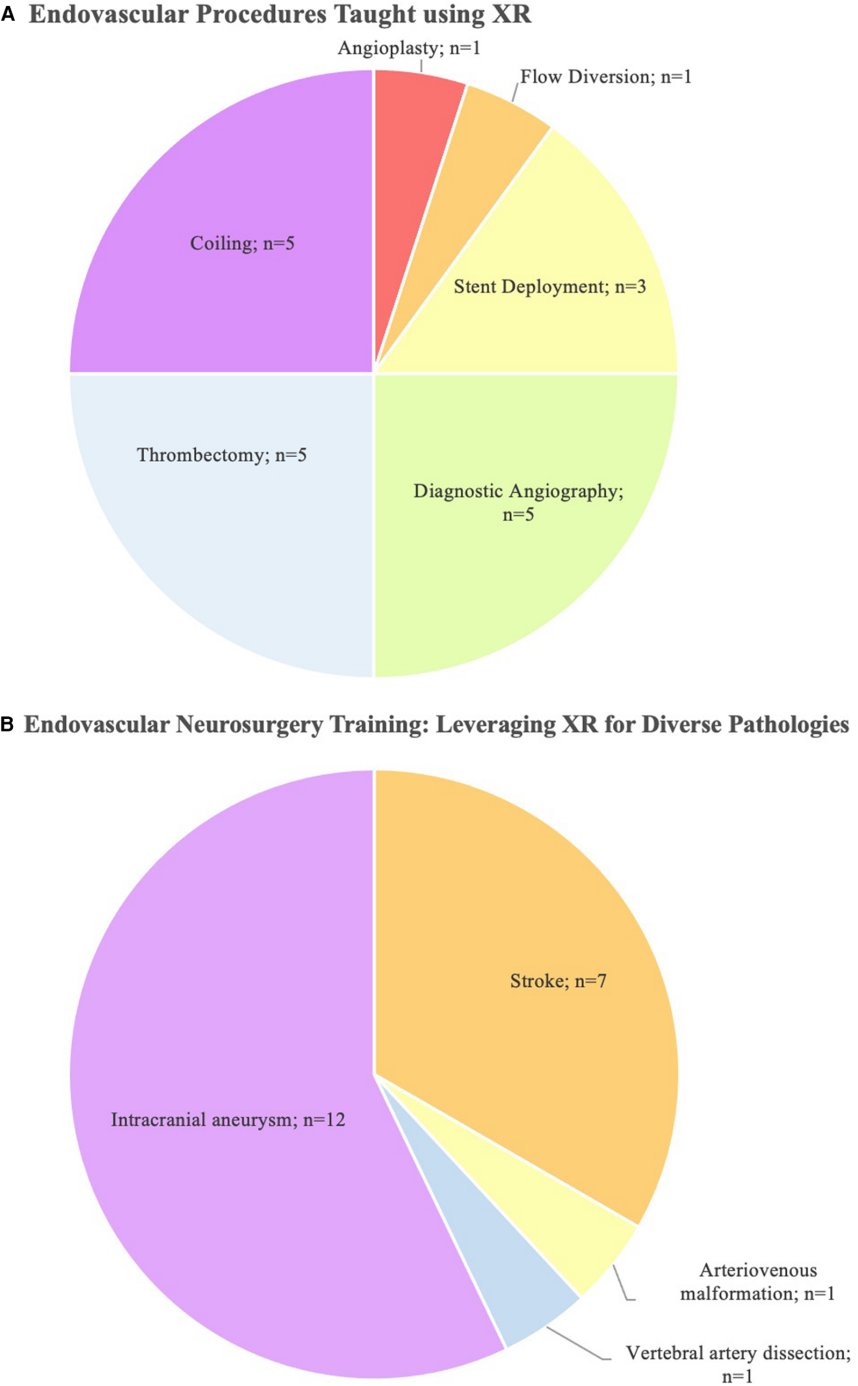
**(A)** A pie chart highlighting the procedural applications of XR in the literature and **(B)** pathologies that were taught with the use of XR. Note that some studies examine multiple procedures or pathologies.

The search strategy identified 408 unique publications, 85 of which underwent full-text review. 19 studies from 2001 to 2023 were included in the final analysis (Figure 1). 13 studies used VR, while 3 studies used MR, and 3 used AR (Table 1). AR was only used for live intra-operative training. MR was only used for live intra-operative training or modeling. VR was used for modeling or simulation (Figure 2). Intracranial aneurysm was the most common pathology that XR was used to teach, followed by stroke (Figure 3A). Coiling (*n* = 5 studies), diagnostic angiography (*n* = 5) and thrombectomy were the most common applications of XR to procedural training (Figure 3B).

**Table 1:** Extended reality in surgical education and training for neurointervention.

Study	XR modality	XR device	Pathology	Procedure	Main findings	Trainee level	Educational application
Briganti et al. (18)	VR	Syngo 3D Aneurysm Guidance Neuro	Intracranial aneurysm	Stent	VR predicted a mean stent diameter of 1.14 mm (0.72 mm; s.d.) and length of 5.56 mm (3.05 mm), but the actual stent diameter and length were 0.19 mm (0.30 mm) and 2.44 mm (2.30 mm)	–	Modeling
Hassan et al. (12, 19)	AR	Proximie	Carotid stenosis, vertebral artery dissection, carotid web, arterial occlusion	Intracranial balloon angioplasty, arterial stenting, diagnostic angiography	AR tele-proctoring platform has shown to successfully guide neuro-endovascular fellows through complex surgical procedures remotely; lower but not statistically significant differences in contrast and fluoroscopy use with AR	Neurointervention resident	Live Intra-operative Training
Schneider et al. (20, 21)	VR	Mentice VIST	Stroke	Thrombectomy	Significant improvements in handling errors, reduction in contrast volume, and fluoroscopy time for thrombectomy	6 attending neurointerventionalists, 4 neurointervention residents	Simulation
Kreiser et al. (22)	VR	Mentice VIST	Intracranial aneurysm	Diagnostic angiography	Beginner surgeons showed significant decreases in total procedure duration, fluoroscopy time and amount of contrast used	4 expert neurointerventionalists, 5 beginner residents, 1 medical student	Simulation
Rai et al. (11)	MR	Magic Leap-1	Intracranial aneurysm	Woven EndoBridge deployment	Significant differences in livestream latency; 0.71 (SD, 0.03) seconds for Wi-Fi, compared to 0.86 (SD, 0.3) seconds for cellular data hotspot	Experienced proctoring neurointerventionalist	Modeling
Deib et al. (4)	MR	Magic Leap 1	Intracranial aneurysm	–	Usage of hand gestures to rotate and inspect aneurysm and surrounding vasculature enhanced trainee performance during procedures	–	Live Intra-operative Training
Elsawaf et al. (23)	VR	Angio Mentor	Intracranial aneurysm, stroke	Diagnostic angiography, coiling, thrombectomy	Use of simulator allows for improvement in angiography/catheter skills and overall improvement in performance metrics for coiling and thrombectomy	Medical student, neurointervention residents	Simulation
Panse et al. (24)	MR	Hololens 2	Intracranial aneurysm	–	Reduced fluoroscopy use with a rotatable 3-D volumetric representation that analyzes gantry approach viability	Attending neurointerventionalist	Modeling
Dardick et al. (25)	VR	Angio Mentor	Intracranial aneurysm, stroke	Coiling, thrombectomy	Decreased time to revascularization in thrombectomy and time to reach key anatomical landmarks in aneurysm coiling	18 neurointervention residents	Simulation
Crossley et al. (26)	VR	Mentice VIST	Stroke	Thrombectomy	Designed VR simulation metrics that could differentiate between expert and novice surgeons	10 attending neurointerventionalists	Simulation
Hussain et al. (27)	VR	NR	Stroke	Thrombectomy	Strongly positive survey results: 29.5/30 (realism), 46/50 (technical issues), 97/110 (teamwork and communication) for intra-professional education	Attending neurointerventionalist, vascular surgery attending, diagnostic radiology attending, anesthesiology attending, and stroke team	Simulation
Ong et al. (28)	VR	HTC Vive	Intracranial aneurysm	–	3-D segmentation was successfully visualized in VR for neurosurgical planning; opaque and translucent modes of visualization highlight unique features	Attending neurointerventionalist	Modeling
Nguyen et al. (5)	VR	Angio Mentor	–	Diagnostic angiography	Strong improvement in novices, who matched procedure time of intermediate surgeons after two rounds	Neurointervention residents	Simulation
Fargen et al. (29)	VR	Mentice VIST	–	Diagnostic angiography	Angiography skills improved significantly through use of hands-on simulator course. Post-course test scores were significantly higher when compared to pre-course	Neurointervention residents	Simulation
Medical Advisory Secretariat (30)	VR	Mentice VIST	Intracranial aneurysm	Coiling	VR can be used not just for assessment in education with metrics. More experienced surgeons had faster procedure times and used less contrast under VR	Attending neurointerventionalists	Simulation
Negoro et al. (31)	VR	Mentice VIST	Intracranial aneurysm	Coiling; stent deployment	Dual-round training model: first training is on VR, second round is using 3-D models	Neurointervention residents	Simulation
Lessard et al. (32)	AR	Prototype	Intracranial aneurysm	Coiling	Guidewire still needs to be manually segmented on a still image; tested on 6 fluoroscopic sequences of 300 images from. Wire Error: 0.19 mm (*σ *= 0.13) laterally and 0.46 mm (σ = 1.75) frontally	–	Live Intra-operative Training
Subramanian et al. (33)	VR	Prototype	Stroke	–	In 3–6 h, generates 3D vasculature from biplane angiograms for preoperative modeling	–	Modeling
Nowinski et al. (9)	AR	NeuroCath	Intracranial aneurysm	–	First design of MR device in endovascular neurosurgery; 3-D and fluoroscopic views as well as haptic feedback	–	Live Intra-operative Training

## 4 Discussion

This systematic review synthesizes the current evidence regarding XR in vascular neurosurgery. We identify several domains of XR technology used to train vascular neurosurgeons across various conditions. In addition, we discuss the opportunities available for clinicians, engineers, and innovators interested in advancing neurosurgical education.

### 4.1 Modeling with XR

XR can be used to generate models of neurovasculature. Interactive modeling is particularly strong at introducing novice surgeons and medical students to neurovascular anatomy. Here, XR models can be generated based on anatomical diagrams, as a supplement–or replacement–to textbooks and cadavers. Many medical schools already use VR or AR to teach anatomy–including but not limited to neuroanatomy–which was particularly useful for remote education during the COVID-19 pandemic (34, 35). While this is well-established, our review demonstrates that XR shows promise not just for early-stage trainees, but also for advanced surgeons. In 2013, a commentary discussed the advantages of augmented fluoroscopy over 2-D biplane angiography. It paves the way for AR in 3-D intra-operative navigation, aiding surgeons in identifying crucial functional brain areas (36). Rather than modeling pre-programmed anatomy, custom XR models can be generated based on a patient's individual anatomy–highlighting morphological differences and anatomical variations. Preoperative planning remains a cornerstone of successful surgical intervention in endovascular neurosurgery. Surgeons can now create patient-specific, three-dimensional models derived from high-resolution imaging modalities, which can be examined and manipulated in XR environments. While pre-programmed models could help medical students develop a general, foundational, anatomical understanding, customizable models help advanced surgeons teach surgical approaches on patients with more complex anatomy. In a comparison of 3-D VR constructions of aneurysms with traditional CTA, 10 neurosurgeons were shown a CTA, provided an assessment and were then shown a 3-D VR construction of the patient's anatomy and asked to reassess the case. In the 26 patients examined, this modality significantly influenced detection of the vascular structure around the aneurysm, ideal head positioning, and neurosurgical approach (37). In 2018, using the HoloLens2 (Microsoft Corporation, Washington, United States), MR was shown to avoid the need for multiple fluoroscopy images by generating a volumetric representation of the vasculature the neurosurgeon can rotate. The device is designed such that the color of the text of the C-arm angle changes based on whether the angle is achievable by the gantry. This interface that can help trainees understand how to approach procedures. In addition, the neurosurgeon can manipulate the system through either hand gestures, eye gaze or voice control (24).

However, there are some technological limitations to customizable models. In 2004, a novel method was developed for translation of biplane angiography into personalized VR 3-D models of patient neurovasculature. While the authors hoped to apply the technology for endovascular neurosurgery in stroke, the VR system still requires up to 6 h to generate a model (33). Recently in 2023, VR was used to assist in stent deployment for anterior circulation aneurysms of 27 patients. While the virtual stent underestimated the length and diameter of the stent, the authors anticipate its application for pre-operative planning with further technological advancement (18). Another study describes the experience using the MxR Magic Leap One (Magic Leap, Florida, United States) for the pre-procedural planning of neurosurgical aneurysm treatment. Similar to the HoloLens 2, hand gestures were used to examine the aneurysm and surrounding neurovasculature; this intuitive user interface highlights an advantage of MR over VR and AR (4).

Customizable AR and VR models have shown clinical promise in small studies. Great progress has been made with the technological capabilities themselves, patient specific models need to be revisited. Future studies should investigate outcomes prospectively and with larger sample sizes. Neurosurgical innovators should also focus on using MR technology over VR and AR for pre-operative planning, due to strengths inherent to the technology's interface.

### 4.2 Live intra-operative and tele-education

In addition to teaching anatomy and explaining cases, we uncover a second key educational role of XR: live intra-operative guidance, where neurosurgeons guide trainees while performing a procedure. Overlays can help a surgeon understand how to approach a procedure–such as the gantry angle feature of the HoloLens 2 described previously (24). In addition to overlays, an important use case of XR is live tele-proctoring, where an experienced specialist can educate a less-experienced surgeon in a remote area on performing a procedure.

Proximie (Proximie, United Kingdom), is an AR platform enabling users to intra-operatively guide trainees. In a series of 10 cases, an attending surgeon used the device to communicate with neurosurgical fellows and provide immediate feedback. In this setting, intra-operative imaging and a video feed of the operating fellow were live-streamed to an attending surgeon in a different geographic location. In addition to a video feed, the surgeon also used AR illustrations to communicate with the trainee in the angiography suite. No complications or deaths were recorded across a variety of procedures, including diagnostic angiograms, balloon angioplasty, and stent deployment. While fluoroscopy time and contrast use were lower compared to in-person supervision, the difference was not statistically significant (12, 19). In Woven EndoBridge device deployment, another AR device called Magic Leap-1 (Magic Leap Incorporated, Florida, United States) guided a trainee over either a cellular data hotspot or a Wi-Fi-based network (11). The minimal latency observed in the study shows promise, especially for education in remote areas or low-income settings with less stable connections to the Internet. XR may enhance surgical training across diverse geographic regions, enhancing collaboration and reducing disparities due to lack of training. However, these resource-limited settings often have little access to novel technologies, and efforts must be made to ensure this is not a barrier as well. However, further studies with much larger sample sizes are needed to demonstrate the long-term benefits of XR for intra-operative neurosurgical education.

Importantly, XR should allow the supervising surgeon to clearly see and understand the actions of the operating trainee, even from a remote location, so advancements in intra-operative education have emphasized improved monitoring technology. A team at Notre Dame Hospital in Montreal, Canada, developed an algorithm for guidewire tracking in endovascular neurosurgery. In this process, the guidewire needs to be manually segmented on a still image before separate algorithms track the lateral and anteroposterior movement. Notably, they achieve a significantly lower error for guidewire tracking than previous papers, and perform the first testing for neurosurgical interventions (32, 38). Gao et al. have proposed a VR system that not only accounts for the displacement of the catheter as it inserts/retracts and rotates but also has haptic feedback when the catheter contacts a blood vessel wall, something a separate commentary from neurosurgeons in 2013 called for (15, 39). Haptic feedback, where touch and physical pressure are electronically transmitted, allows for a more intuitive experience for the surgeon. By augmenting XR with the sensation of touch, technical advancements in endovascular tool-artery interaction paved the way for haptic feedback technology in neurointervention. In 2007, an endovascular neurosurgery-specific catheter-artery interaction database was reported, pioneering haptic feedback technology (40). Additionally, a VR device has been developed called the NeuroCath (Neuroradiology Catheterization Simulator, Poland) that provides 3-D fluoroscopic as well as haptic feedback (8, 9, 41). In addition to haptic feedback, another key augmentation is multiple visualization modes; a team at Johns Hopkins Hospital report how the HTC Vive (New Taipei City, Taiwan) provided an opaque display mode that was helpful to evaluate morphological parameters, while a translucent projection was useful when evaluating inflow and outflow tracts or visualizing overlapping anatomical structures (28).

Clinical studies examining XR in live education are promising and show strong technological foundations. Further study is warranted because of a large potential to improve the quality of neurosurgical education globally and reduce disparities due to poor training/clinical exposure. Notably, clinical studies on existing devices all examine only AR, while designs focus on VR devices–an important niche for future MR research to fill (Figure 2). We note that all studies examining live intra-operative education have an experienced surgeon use XR to guide an inexperienced one. However, we propose that XR technology could also be used in the opposite way: to have novice surgeons to virtually observe more experienced surgeons. Operative videos are popular in leading neurosurgical journals, but XR technology may provide a superior, more immersive, experience than videos. Recently, a team at Mount Sinai Hospitals in New York published a VR arteriovenous malformation resection (42). We hope to see further clinical studies comparing operative videos with XR experiences in the coming years.

### 4.3 Neurosurgical simulation

Lastly, we address the use of XR in simulations and whether it provides a promising educational approach that improves surgical performance. Several reviews and commentaries discuss using XR to teach neurosurgical procedures through simulation (3, 6, 43–47).

The most frequently identified platform to aid in neurosurgical simulation was the Mentice Vascular Intervention Simulation Trainer (Figure 2). The Mentice Vascular Intervention Simulation Trainer (VIST; Mentice, Sweden) is a metric-based VR simulator that is able to accept real materials, including catheters, coils, and stents, to assess trainee skills and rehearse procedures (31). Neuroradiologists who trained with the Mentice VIST improved operative speed and reduced contrast use in the simulation of cerebral aneurysm embolization (30). Kreiser et al. found that the Mentice VIST decreased total procedure duration and fluoroscopy time during diagnostic angiography simulations, but only in inexperienced beginner surgeons (22). In contrast, among interventional neuroradiology attendings and residents in mechanical thrombectomy simulations, utilizing the Mentice VIST improved handling errors, reduced contrast volume, and reduced fluoroscopy time. Each neuroradiologist in this study reported positive attitudes toward the simulator (20, 21).

In addition to the Mentice VIST, the Angio Mentor (Simbionix Surgical Science, Sweden), a commercially available simulation learning platform, improves a mentor's ability to provide haptic feedback following simulated endovascular procedures with catheter-based systems and enhances trainees' understanding of cerebrovascular anatomy (23). In endovascular neurosurgery trainees, the Angio Mentor has decreased time to every procedural benchmark for simulated revascularization of a middle cerebral artery thrombosis (25). This platform has demonstrated similar results in diagnostic cerebral angiogram simulations (5). Simulations using the Magic Leap 1 and Snygo 3-D showed educational benefits when simulations were performed across a variety of device types, including the Woven EndoBridge (MicroVention, California, USA), flow diverter P64, the flow diverter P48 (Phenox, Germany), and the pipeline embolization device (Medtronic, California, USA) (11, 18).

Unexpectedly, XR in endovascular neurosurgery was useful to non-surgical specialties as well. A team of vascular surgery, anesthesiology, and radiology team members were led by a neurosurgeon in drafting carotid artery stenting protocols. These protocols were tested in a VR environment, and participants reported an increased understanding of what to do during stent deployment, and another similar study reported near-perfect survey scores from participants in the categories of realism, technical issues, teamwork, and communication (27, 48).

VR technology from ImmersiveTouch (Illinois, USA) has shown promise in ventriculostomy, lumbar puncture, and trigeminal rhizotomy simulations, but have not published results on vascular neurosurgical procedures. Notably, the team behind this project has received almost half a million dollars in grant funding for their work examining a VR haptic model of an MCA bifurcation aneurysm from the United States National Institutes of Health. This information is publicly available to anyone by accessing the National Institutes of Health grant reporter page (49).

**Figure 4:**
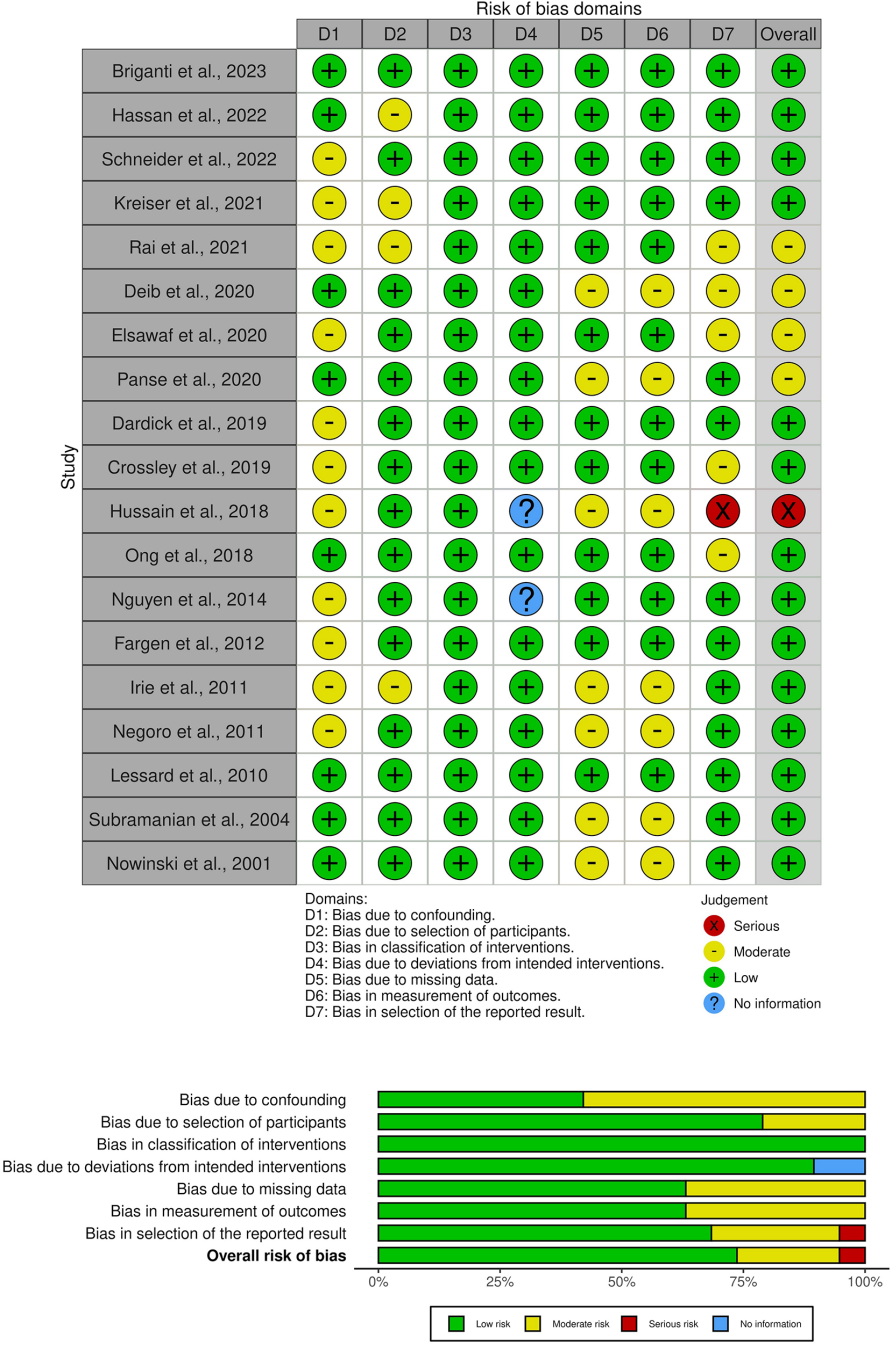
A risk-of-bias visualization was generated using robvis: An R package and shiny web app for visualizing risk-of-bias assessments. Studies on the left axis are classified into major sub-topics.

Despite the apparent benefits of augmented reality (AR) and virtual reality (VR) in endovascular neurosurgery, a detailed review and formal risk-of-bias assessment using ROBINS-I revealed several opportunities for growth in the current evidence base (Figure 4). There is substantial heterogeneity in the XR outcome measures reported, which range from subjective user satisfaction scores to objective measures of task performance. Most importantly, we identify a gap in the literature on long-term outcomes, limiting our understanding of the lasting impact and retention of skills or benefits imparted by XR. In addition, whether this technology is cost-effective should be examined.

## 5 Conclusions

This systematic review synthesizes the current evidence regarding the use of XR technology in vascular neurosurgery, identifying various domains where XR is employed to train surgeons and highlighting opportunities for future innovation. While the role of XR in teaching anatomy is well-established for medical students and junior trainees, we reveal that XR technology offers significant benefits for advanced surgeons in preoperative planning through the creation of patient-specific, three-dimensional models derived from high-resolution imaging modalities. These customizable models, which can be manipulated in XR environments, provide critical insights into individual anatomical variations, enhancing the precision of surgical interventions. However, technological limitations persist, such as the lengthy time required to generate simulations and the occasional inaccuracies in patient-specific models. Despite these technological barriers, preliminary studies demonstrate the clinical promise of XR, suggesting its potential to transform neurosurgical training and planning.

In addition to pre-operative education, XR shows great potential in live intra-operative guidance, where experienced surgeons can remotely oversee and instruct trainees. Platforms like Proximie and the Magic Leap-1 have facilitated remote guidance with minimal latency–even over cellular data hotspots–which could prove especially valuable in resource-limited settings. However, we call for further large-scale studies to substantiate these benefits and explore new applications, such as our novel proposal to use XR to allow novice surgeons to virtually observe experienced colleagues.

Finally, VR simulations, like the Mentice VIST and Angio Mentor, have demonstrated efficacy in improving procedural performance and reducing training times. These tools also show promise in enhancing interdisciplinary education. While AR and VR simulation consistently show benefits across many studies and diverse metrics, there remains a need for rigorous evaluation of XR's impact on long-term skill acquisition. Reports on long-term outcomes, implementation in low-resource settings, technological improvements, and cost-effectiveness are still needed, but augmented, mixed, and virtual reality together hold promise for a paradigm shift in endovascular neurosurgery training.
