# Supplementary material for: Advancing endovascular neurosurgery training with extended reality: opportunities and obstacles for the next decade
Source: Front Surg. 2024 Aug 27;11:1440228. doi: 10.3389/fsurg.2024.1440228 (PMC11385296; doi:10.3389/fsurg.2024.1440228)
Supplement: Supplementary file 1 [file Table1.docx]

**Supplemental Data 1**

The following search terms were used.

1. PubMed

((endovascular neurosurgery[Title/Abstract]) OR (neuroendovascular[Title/Abstract]) OR (neuro-endovascular[Title/Abstract]) OR (interventional neurorad*[Title/Abstract]) OR (neurointervention*[Title/Abstract]) OR (neuro-intervention*[Title/Abstract]) OR (neuro intervention*[Title/Abstract])) AND ((augmented reality[Title/Abstract]) OR (AR[Title/Abstract]) OR (virtual reality[Title/Abstract]) OR (VR[Title/Abstract]))

2. Scopus

TITLE-ABS-KEY(((endovascular AND neurosurgery) OR (neuroendovascular) OR (neuro-endovascular) OR (interventional AND neurorad*) OR (neurointervention*) OR (neuro-intervention*) OR (neuro AND intervention*)) AND ((augmented AND reality) OR (ar) OR (virtual AND reality) OR (vr)))

3. EMBASE

('endovascular neurosurgery'/exp OR 'endovascular neurosurgery' OR neuroendovascular OR 'neuro-endovascular' OR 'interventional neurorad*'/exp OR 'interventional neurorad*' OR neurointervention* OR 'neuro-intervention*' OR 'neuro intervention*') AND ('augmented reality' OR AR OR 'virtual reality' OR VR)

4. Web of Science

TS=((endovascular neurosurgery) OR (neuroendovascular) OR (neuro-endovascular) OR (interventional neurorad*) OR (neurointervention*) OR (neuro-intervention*) OR (neuro intervention)) AND TS=((augmented reality) OR (AR) OR (virtual reality) OR (VR))

5. OVID

((endovascular neurosurgery or neuroendovascular or neuro-endovascular or interventional neurorad* or neurointervention* or neuro-intervention* or neuro*intervention*).ti,ab,kw.) and ((augmented reality or AR or virtual reality or VR).ti,ab,kw.)
